# Special Issue ‘Advances in Neurodegenerative Diseases Research and Therapy 2.0’

**DOI:** 10.3390/ijms25094709

**Published:** 2024-04-26

**Authors:** Sumonto Mitra

**Affiliations:** Division of Clinical Geriatrics, Center for Alzheimer Research, Department of NVS, Karolinska Institutet, 141 52 Huddinge, Sweden; sumonto.mitra@ki.se

Neurodegenerative disorders (NDs) and the development of various therapeutic strategies to combat them have received increased attention in recent decades [1]. This is primarily due to rapid aging in populations worldwide [2]; aging is known to be the primary risk factor for various NDs [3]. In 2015, around 1 billion people were above 60 years of age (~12% of world’s population), and this figure is estimated to double by 2050 to around 2.1 billion (~22% of world’s population), among which 80% of these individuals would be in low- and middle-income countries [2]. Presently, major high-income countries are already facing increasingly aging populations, where the average age is approaching or already exceeds 40 years [4]. Thus, there is an increasing need to effectively manage NDs and develop therapies to meet future healthcare demands.

NDs are often described as conditions that damage the central or peripheral nervous system, and can be broadly categorized as the dementia type (Alzheimer’s disease—AD; frontotemporal dementia—FTD; chronic traumatic encephalopathy—CTE; Lewy body dementia—LBD; and limbic-predominant age-related TDP-43 encephalopathy—LATE), demyelinating diseases (multiple sclerosis—MS; neuromyelitis Optica spectrum disorder—NMOSD), the Parkinsonism type (primarily Parkinson’s disease, PD, or Parkinson-like disorders), motor neuron diseases (amyotrophic lateral sclerosis—ALS; progressive supranuclear palsy—PSP), and prion diseases (Creutzfeldt–Jakob disease—CJD). Often, some features may overlap between different NDs, complicating diagnostic and therapeutic approaches [5].

Dementia-type disorders are the most prevalent form of ND; they affected over 55 million people worldwide during 2020, among whom AD accounted for 60–70% of all cases, and this figure is likely to double by 2050 [6,7]. Although diagnostic markers for various NDs are available, formal diagnoses of NDs are often not successfully achieved [8], thereby reducing access to available treatments. In various cases, therapeutic strategies need to be improved and implemented to benefit society at large [1]. Some of the major obstacles in the field of ND therapy and disease management include limited therapeutic options, especially due to ineffective drug delivery methods; unknown etiological factors; and complications from other concurrent pathological conditions [8,9,10]. The research work presented in this Special Issue encompasses various aspects of these obstacles in ND research and presents novel aspects of drug delivery [11], molecular mechanisms associated with ND etiologies [12,13], the molecular pathways shared between diseases [14,15], and therapeutic approaches [16,17].

The delivery of drugs to intended locations and at optimum levels has been a major issue for the development of effective therapies to treat various NDs [9,10]. Due to the limitation imposed by the blood–brain barrier (BBB), the entry of larger molecules across the BBB has been previously facilitated by targeting the transferrin receptors [18]. In this issue, Choi et al. [11] utilized transferrin-conjugated melittin-loaded L-arginine-coated iron-oxide nanoparticles (Tf-MeLioNs) to demonstrate their capacity to deliver drugs to brain tissue in 5XFAD transgenic mice and reduce AD-specific pathological markers. More importantly, they showed that by encapsulating cytolytic drugs like melittin within a nanocarrier, their peripheral toxicity is reduced and targeted delivery to brain tissue increases. This approach may be helpful in reducing and refining dosages to achieve clinically relevant effects for various drugs while reducing off-target effects.

Traditionally, it has been difficult to treat a disease without proper knowledge of the underlying etiologies. In view of this, Sheu et al. [12] and Jia et al. [13] reported novel underlying molecular changes occurring in different situations within NDs. Although the BBB has been associated in protecting the brain from peripheral insults, BBB disruption occurs during various stages of different NDs [19], and this may allow molecules like thrombin to invade brain tissue. Sheu et al. show that microglial activation mediated by thrombin exposure could lead to neuronal death and neurobehavioral alterations in rats, and validated the role of HMGB1 (high-mobility group box chromosomal protein 1) using in vivo and in vitro approaches. These studies highlight that HMGB1 could be a potential target with which to counter neuro-inflammation and eventually to reduce neuronal degeneration. Similarly, Jia et al. elucidate the interplay between iron and LRRK2 (Leucine-rich repeat kinase 2) in inducing the death of dopaminergic neurons and provide molecular reasoning for the occurrence of sporadic and familial forms of PD linked to LRRK2 mutations and altered activity. Jia et al. also show that iron plays an important role in modifying LRRK2 activity, which in return increases iron accumulation further in the substantia nigra of brain, explaining the iron deposition observed in PD. In combination, these two studies highlight the molecular alterations that can serve as therapeutic targets in managing NDs in the future.

It has been widely acknowledged that various NDs may show overlapping pathological markers [3,6,7,9]. In this issue, Shi et al. [14] develop this idea and map the common and dissimilar pathways between NDs and infection from the novel severe acute respiratory syndrome coronavirus-2 (SARS-CoV-2). This is an important aspect since several reports have revealed the neurological and psychological effects of SARS-CoV-2 and that infected patients are predicted to have a higher disposition to suffering from diverse NDs [20,21]. Due to the worldwide infection rates of SARS-CoV-2 and aging population trends, complications in terms of ND cases and their management can be observed in different societies. Conversely, a review report from Sun et al. [15] summarizes the function and contribution of presenilin, a transmembrane protein primarily known for its role in AD, and highlights its multi-functional role in various other NDs. Although presenilin has been a highly researched drug target in AD due to its role in the production of amyloid beta, this review highlights that presenilin has many other crucial biological functions. Thus, inhibiting presenilin as a form of AD therapy may have several side effects. This information holds potential in gauging the efficacy of therapeutic targets and in minimizing off-target effects.

Finally, this Special Issue presents therapeutic strategies through an original report by Seo et al. [16] and a narrative review by Cutuli et al. [17]. Utilizing genetic modification approaches and in vivo model systems, Seo et al. demonstrate the development of a specific promoter-driven gene expression in the neurons expressing DOPA decarboxylase (DDC) at substantia nigra pars compacta (SNpc). They further characterize the therapeutic efficacy of their construct via the chemogenetic activation of DDC-positive neurons at SNpc, which relieve PD-related motor symptoms and rescue the expression of dopaminergic neuron markers. Considering the results together, Seo et al. develop an effective strategy to modulate the gene expression of DDC-positive neurons as a form of therapy for PD. On the other hand, Cutuli et al. review the connection between physical exercise, gut microflora, inflammatory pathways, and their communication with brain-associated pathways in the context of AD. Cutuli et al. present various factors that link physical exercise, gut microbiota, and AD, and argues that they can affect each other in considerable ways. They propose that physical exercise is an important component in the regulation of gut microbiota, and can modulate brain-associated pathways in turn, sometimes through inflammatory processes, neurotransmitter regulation, or neurotrophin regulation, among many other mechanisms. These connections are topics of ongoing intense research and have also been considered in the design of new clinical trials for AD, for example the FINGER trials [22], which are now actively participated in across various countries.

This Special Issue is a great example that highlights all the various novel efforts in ND research and therapy development, which range across various disciplines. Developing a better understanding of diseases, choosing the right druggable target and finally employing an optimized drug delivery regime require coherent efforts and pave the path for future work towards developing efficient therapies against NDs.
